# Two TonB-dependent outer membrane transporters involved in heme uptake in *Anabaena* sp. PCC 7120

**DOI:** 10.15698/mic2024.01.812

**Published:** 2024-01-09

**Authors:** Julia Graf, Martin Schöpperle, Rafael Pernil, Enrico Schleiff

**Affiliations:** 1 Institute for Molecular Biosciences, Goethe University Frankfurt, Max von Laue Str. 9, 60438 Frankfurt, Germany.; 2 Current address: Lonza Cologne GmbH, Köln, Germany: mschoepperle@gmail.com; 3 Frankfurt Institute for Advanced Studies, Ruth-Moufang-Straße 1, 60438 Frankfurt, Germany.; 4 Buchmann Institute for Molecular Life Sciences, Max von Laue Str. 11, 60438 Frankfurt, Germany.

**Keywords:** cyanobacteria, metal uptake, metal stress, iron, protoporphyrin IX, chlorophyll *a*

## Abstract

Low availability of micronutrients such as iron has enforced the evolution of uptake systems in all kingdoms of life. In Gram-negative bacteria, outer membrane, periplasmatic and plasma membrane localized proteins facilitate the uptake of iron-loaded chelators, which are energized by TonB proteins. The specificity of different uptake systems likely depends either on the endogenously produced siderophore or on the bioavailability of iron-chelator complexes in the environment. Hence, an uptake system for schizokinen produced by the model cyanobacterium *Anabaena* sp. PCC 7120 exists, while bioinformatics analysis suggests the existence of additional systems, likely for uptake of xenosiderophores. Consistently, proteins encoded by *alr2153* (*hutA1*) and *alr3242* (*hutA2*) are assigned as outer membrane heme transporters. Indeed, *Anabaena* sp. PCC 7120 can utilize external heme as an iron source. The addition of heme resulted in an induction of genes involved in heme degradation and chlorophyll *a* synthesis and in an increase of the chlorophyll *a* content. Moreover, iron starvation induced the expression of *hutA1*, while the addition of heme led to its repression. Remarkably, the addition of a high concentration of heme but not iron starvation resulted in *hutA2* induction. Plasmid insertion mutants of both genes exhibited a reduced capacity to recover from iron starvation by heme addition, which indicates a dependence of heme uptake on functional HutA1 and HutA2 proteins. The structural model generated by bioinformatics methods is further in agreement with a role in heme uptake. Thus, we provide evidence that *Anabaena* sp. PCC 7120 uses a heme uptake system in parallel to other iron acquisition systems.

## INTRODUCTION

Iron availability is known to determine the growth of phytoplankton in aquatic environments [1, 2]. Iron is of high importance for cyanobacterial growth due to the existence of iron-containing proteins in the photosynthetic apparatus and in enzymes of the nitrogen fixation machinery [3]. As a consequence, the intracellular concentration of iron in cyanobacteria is one order of magnitude higher than in *Escherichia coli* [4]. The high iron demand stands in contrast to the availability of iron in aquatic environments.

Several dissolved iron species exist in marine systems, which are all only present in the nanomolar to subnanomolar range [5]. Moreover, about 99% of the iron is bound to various organic ligands [6–9]. Considering the low availability of iron, aquatic organisms resorted to different mechanisms to use the various iron species to be able to adapt to the constantly changing conditions and iron availabilities in the ecosystem. One example for the sequestering of iron are siderophores, which are low molecular weight organic compounds that are able to chelate iron with high affinities [10]. They are known to be secreted by species of all domains and kingdoms of life [11]. Siderophore synthesis and secretion is in general linked to iron availability and increases with iron limitation [12].

The acquisition of substrates that exceed the diffusion size limit for porins or that exist in low quantities in the environment like iron-siderophore complexes is dependent on active TonB-dependent transport systems [13, 14]. TonB-dependent transporters (TBDT) are embedded in the outer membrane. They consist of a 22-stranded β-barrel protein with an N-terminal globular plug domain and a TonB box [15]. Additionally, the Ton system is embedded in the plasma membrane and consists of the proteins ExbB, ExbD and TonB, which interacts C-terminally with the TonB box of the TBDT [16]. The transport of the siderophore relies on the electrochemical potential across the plasma membrane and the energy is transmitted to the TBDT through the TonB protein [14]. Upon substrate binding by the TBDT, which is thought to be energy independent, conformational changes are induced to allow transport across the outer membrane.

Another way of iron acquisition that follows similar uptake principles as the ones of siderophores is uptake of heme. Besides its importance as iron source, heme acquisition was found to be crucial for the survival and virulence of bacterial pathogens [17]. Heme belongs to the porphyrins that are characterized by a tetrapyrrole ring, which permits the complexation to different metal cations [18, 19]. Heme is involved in important biochemical functions such as photosynthesis, respiration and nitrogen fixation [4]. Apart from being a substrate for the binding of iron, porphyrins are important in oxygen transport, storage and reduction as well as electron transfer and hydrogen peroxide utilization [20]. In cyanobacteria heme is also an essential intermediate, e.g., for the synthesis of phycobilins [21]. However, heme concentrations within the cell have to be regulated as heme catalyzes the formation of reactive oxygen species resulting in cellular damage and oxidative stress [22, 23].

The biosynthesis of heme depends strongly on iron availability [24–27]. The synthesis of heme and chlorophyll *a* up to protoporphyrin IX follow the same pathway. Subsequently, heme is synthesized from protoporphyrin IX by Fe^2+^ insertion whereas Mg^2+^ insertion is the first step to the chlorophyll *a* pathway. Besides synthesis, some bacteria can utilize external sources of heme. The principals of the uptake of external heme by different Gram-negative bacterial pathogens can take up free heme or heme-containing proteins like hemoglobin (Hb) or hemopexin [17]. Moreover, pathogens such as *Pseudomonas aeruginosa* contain heme-uptake systems [28]. The system involves one outer membrane heme receptor (PhuR) of the TBDT family, a periplasmic heme-transport protein (PhuT) and inner membrane proteins (PhuUVW, [28]).

The model organism *Anabaena* sp. PCC 7120 (hereafter *Anabaena*) is a heterocyst-forming filamentous cyanobacterium. A filament comprises individual cells surrounded by plasma membrane and a peptidoglycan layer. The outer membrane is, however, continuous along the whole filament, which might consist of hundreds of cells [29]. In the genome of *Anabaena* 22 putative TBDTs were found by bioinformatic analysis [30, 31]. Four of these TBDTs are already experimentally characterized, namely the iron and copper transporter IacT [32] and three proteins that are part of the schizokinen uptake system [4]. The latter are homologs of the hydroxyl-carboxylate siderophore transporter IutA [33] and are annotated as SchT (Alr0397), IutA1 (Alr2209) and IutA2 (Alr2581) in *Anabaena* [34–36]. They are required for the uptake of the siderophore secreted by *Anabaena* [37].

The other 18 TBDTs in *Anabaena* have been assigned by bioinformatics analysis, e.g., as heme transporting TBDTs [30], but their function has not been experimentally confirmed. Two of them were previously assigned as HutA-like [30] based on sequence characteristics, and we extend the annotation to HutA1 encoded by *alr2153* and HutA2 encoded by *alr3242*. The insertional mutants of *alr2153* (*hutA1*) and *alr3242* (*hutA2*) did not show any alteration of uptake of Fe-schizokinen and Fe-aerobactin when compared to wild-type [36]. Interestingly, although heme uptake by cyanobacteria has not been experimentally established, about 30% of all cyanobacteria code for HutA-like proteins in their genome [4]. Consequently, heme uptake by *Anabaena* and the function of the proteins encoded by *alr2153* (*hutA1*) and *alr3242* (*hutA2*)*,* namely HutA1 and HutA2, respectively, was explored. We demonstrate that *Anabaena* can use external heme as iron source. Moreover, we present gene expression analysis evidence for a relevance of the two individual proteins under different environmental conditions. The phenotype analysis of mutants generated by plasmid insertion of these genes suggests a function of the two TBDTs in heme uptake.

## RESULTS

### *Anabaena* utilizes heme as iron source

**Figure 1:**
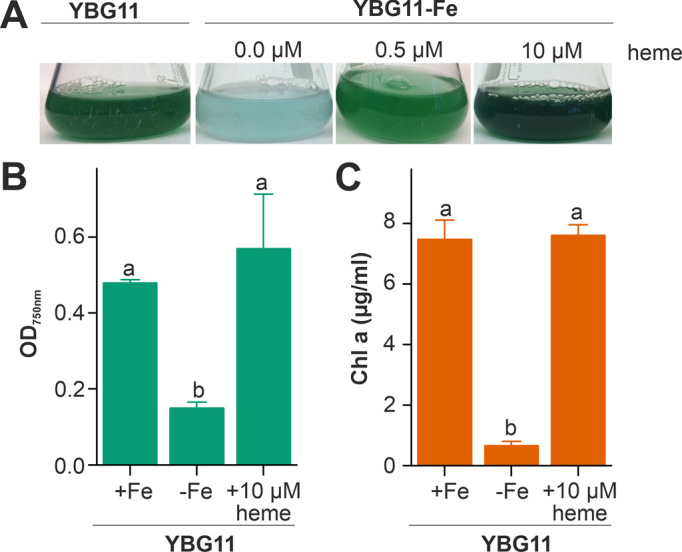
FIGURE 1: Heme utilization by *Anabaena.* **(A)** *Anabaena* was grown in YBG11 (left), YBG11 without iron (second), YBG11 without iron but with 0.5 µM heme (third) or 10 µM heme (right). 50 ml cultures were inoculated to OD_750 nm_ = 0.02 and previously grown in YBG11 medium. Images were taken seven days after inoculation and representatives of multiple replicas are shown. **(B, C)** The optical density (B) or the chlorophyll *a* content (C) of *Anabaena* grown as in (A) was measured and is represented as average of n≥4 experiments with indicated standard deviation. In (B) and (C) the statistical analysis was performed using ANOVA with ranks indicated with p<0.05.

The capacity of *Anabaena* to utilize heme as an iron source was analyzed by comparison of the growth in medium without iron after supplementation with heme as the only iron source. The growth was compared to the growth in standard YBG11 medium as well as in iron-depleted YBG11 medium. The culture changed from green to cyan color in response to iron starvation, because of the decrease of chlorophyll *a* (**Figure 1A**). Moreover, the culture growth was recovered when the iron-depleted YBG11 medium was supplemented with 0.5 and 10 µM heme (**Figure 1**). Further, the growth in the absence of iron was reduced and after seven days the culture density was 30% of that in the presence of normal iron sources (**Figure 1B**). The growth in the absence of iron was restored when 10 µM of heme was added to the medium (**Figure 1B**). Consistent with a recovery from starvation, the chlorophyll *a* content, used as marker for iron starvation [35], decreased 10-fold when *Anabaena* was grown in iron depleted medium, but not when grown in the iron-free medium supplemented with 10 µM heme (**Figure 1C**). This result indicates that *Anabaena* is able to use heme as an iron source, either directly as a metabolite or by release of iron through decomposition of heme.

### Heme and chlorophyll *a* synthesis pathways are regulated by iron and heme availability

**Figure 2:**
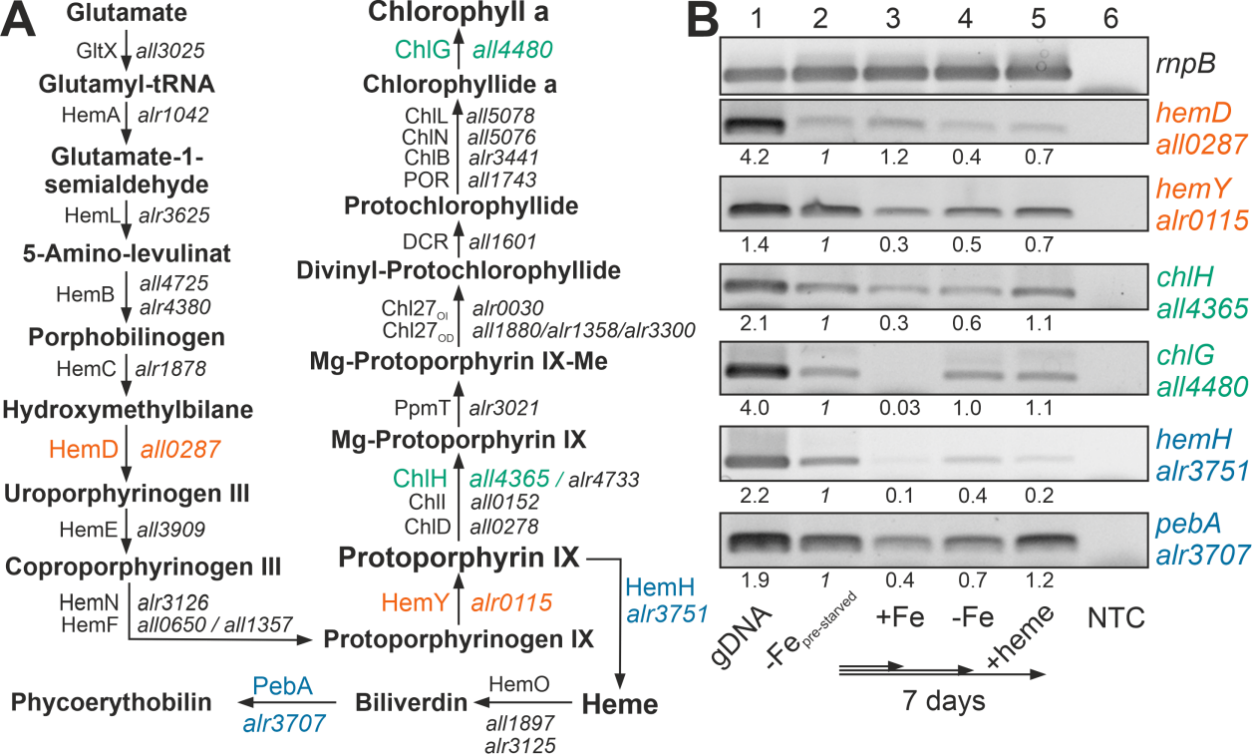
FIGURE 2: Expression of genes involved in chlorophyll *a* and heme synthesis after iron starvation or heme supply. **(A)** The synthesis pathway of heme and chlorophyll *a*, as well as the first steps of the heme degradation pathway, are shown. Intermediate metabolites, proteins involved and the gene coding for these proteins in *Anabaena* are indicated. Orange color indicates genes of the common pathway analyzed; green, genes analyzed of the chlorophyll *a* synthesis; blue, genes analyzed of the heme metabolism. HemF: O_2_-dependent coproporphyrinogen oxidase; HemN: O_2_-independent coproporphyrinogen dehydrogenase, CHL27_OD_/CHL27_OI_: aerobic/anaerobic magnesium-protoporphyrin IX monomethyl ester cyclase. Note: *hemN* and *hemO* are transcribed in a transcriptional unit; ChiL, ChiN and ChlB form a complex. **(B)** RNA was isolated from pre-starved *Anabaena* (lane 2) transferred for seven days to YBG11 (lane 3), YBG11 without iron (lane 4) or YBG11 without iron but 30 µM heme (lane 5). RT-PCR was performed using oligonucleotides for amplification of the indicated genes. The efficiency of the primer was controlled on isolated gDNA (lane 1), and RNA isolation was probed by RT-PCR without reverse transcriptase (NTC, lane 6). Amplification of *rnpB* is shown as control. The intensity of the bands shown was quantified with ImageJ, normalized to the expression of the genes in the pre-starved cultures and shown for guidance.

The recovery of the chlorophyll *a* level after heme addition prompted the analysis of the expression of the chlorophyll *a* synthesis pathway by RT-PCR (**Figure 2A**). All genes analyzed were expressed in pre-starved cultures by growth for 21 days in the absence of iron (**Figure 2B**, lane 2) and most of the genes had a comparable or even reduced mRNA level upon prolongation of starvation for additional seven days (lane 4). After transfer of the pre-starved cultures into YBG11 with iron, all genes were reduced in expression after seven days of growth, except of *hemD*, which remained stable (lane 3).

The presence of 30 µM heme for seven days did not result in a decline of the expression of the genes involved in chlorophyll *a* synthesis in comparison to the pre-starved cultures (**Figure 2B**, lane 5). However, the expression of *hemH*, which is required for the synthesis of heme from protoporphyrin IX, was largely reduced in the presence of external heme (lane 2 vs. 5), and the level of expression of the gene encoding the enzyme involved in the decomposition of heme (*pebA*) was 3-fold higher when compared to cultures grown in the presence of iron (lane 3 vs. 5).

Thus, comparing pre-starved cultures (**Figure 2B**, lane 2) and cultures supplemented with iron (lane 3) suggests that iron starvation results in an enhanced expression of the tested genes except of *hemD*. This is consistent with the observed dependence on FurA of the genes of the heme synthesis pathway [38]. In contrast, prolonged starvation repressed the conversion of protoporphyrin IX to heme by reduction of the mRNA level of *hemH* (lane 2 vs. 4). The addition of heme resulted in repression of the endogenous heme synthesis by a reduced expression of *hemH*, which was similar to the level in the presence of iron (lane 3 vs. 5). More importantly, iron starvation as well as addition of heme enhanced the expression of *pebA* (lane 3 vs. 2 or 5). This suggests that iron-loaded heme will likely be decomposed to release iron for other pathways during iron starvation or by addition of heme.

### Expression of *hutA1* and *hutA2* is iron- and heme-supply sensitive

**Figure 3:**
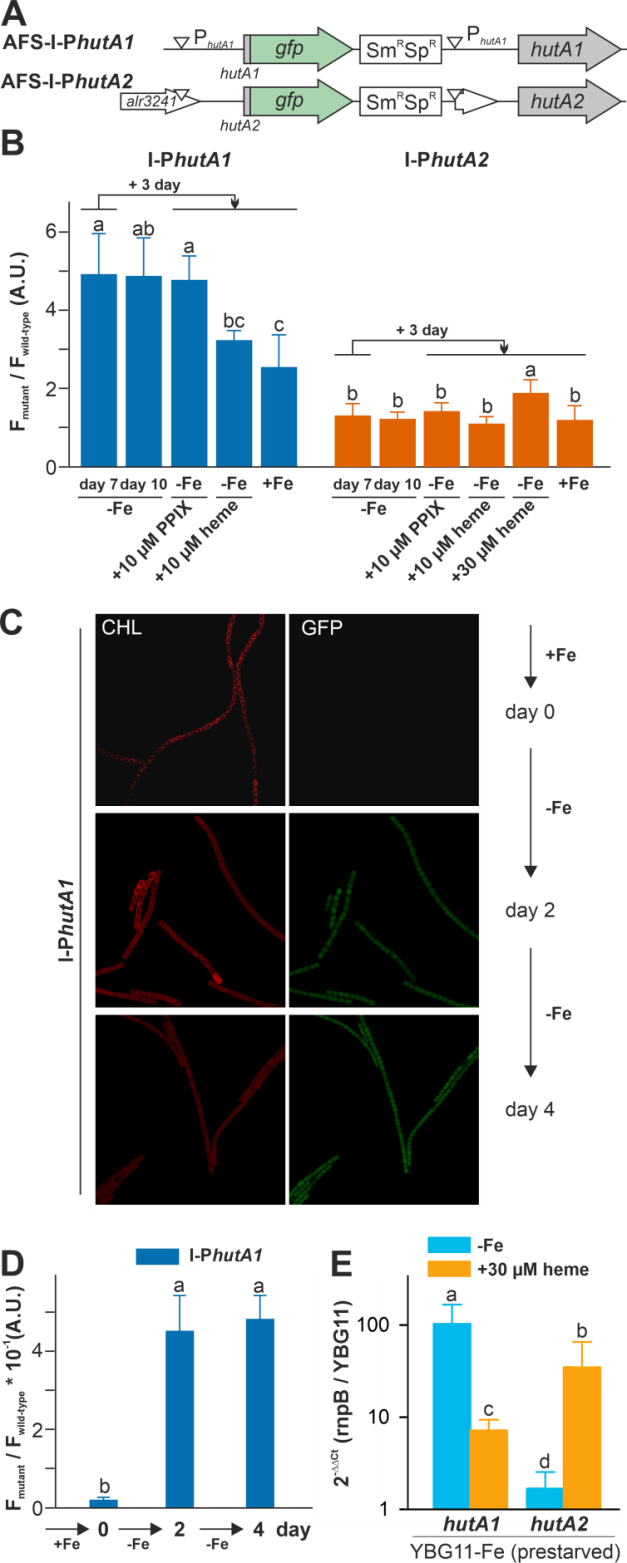
FIGURE 3: *hutA1* and *hutA2* are expressed in iron- and heme-dependent manner. **(A)** Strategy for genomic plasmid insertion to generate AFS-I-P*hutA1* and AFS-I-P*hutA2.* The white downward triangles indicate the predicted promoter start sites. **(B)** *Anabaena* wild-type, AFS-I-P*hutA1* (left) and AFS-I-P*hutA2* (right) strains were grown in YBG11 medium without iron for seven days (first bar). After seven days the culture was transferred into a new medium for growth for three days in YBG11-Fe (second bar), YBG11 medium without iron but with 10 µM protoporphyrin IX (third bar), 10 µM (forth bar) or 30 µM heme (fifth bar for AFS-I-P*hutA2*) or in YBG11, which contains iron (last bar). The ratio of the GFP fluorescence of the mutant strain and the background in wild-type *Anabaena* is shown. The results represent the average of at least three independent experiments. **(C)** AFS-I-P*hutA1* was grown in YBG11-Fe medium cultures (OD_750 nm_ = 0.02) supplemented with 10 mM NaHCO_3_ and bubbled with CO_2_-enriched air (1% CO_2_ v/v). Growth was monitored on day 0 (top), day 2 (middle) and day 4 (bottom). Images are representative for multiple replicas and autofluorescence (left) and GFP fluorescence (right) are shown. **(D)** Images of *Anabaena* wild-type and AFS-I-P*hutA1* strains were taken from (C) and the GFP fluorescence was quantified using a cross section of representative cells. The results represent the average of five independent cultures. **(E)** *Anabaena* pre-starved for 21 days was transferred to YBG11 medium, YBG11 medium without iron or YBG11 medium without iron but with 30 µM heme. After three days of incubation, RNA was extracted and qRT-PCR was performed. Ct values for *rnpB* and for the indicated gene in cultures grown in YBG11 medium were used to calculate the presented values. The average and standard deviation of n>3 independent biological replicas are shown. The statistical analysis in (B), (D) and (E) was performed using ANOVA with ranks indicated with p<0.05.

Considering that *Anabaena* can utilize heme (**Figure 1**), the expression of the two genes, *hutA1* (*alr2153*) and *hutA2* (*alr3242*) was analyzed. The two genes coded for HutA-like TBDTs as previously proposed based on bioinformatics analysis [30]. Fusions of the green fluorescent protein (GFP) to the promoter regions of *hutA1* and *hutA2* were generated. These fusions contain the start codon of the respective gene and are under the control of the endogenous promoters. The constructs were transferred into *Anabaena* to generate the strains AFS-I-P*hutA1* and AFS-I-P*hutA2* (**Figure 3A**; **Table 2**). The genome insertion was confirmed, while we did not probe for segregation (data not shown).

Both strains were pre-starved by growth in iron-depleted medium for seven days. Subsequently, cell cultures were inoculated in iron-depleted medium, iron-depleted medium supplemented with 10 µM protoporphyrin IX (PPIX) representing the last precursor in heme synthesis, 10 µM of heme or standard medium containing iron, and grown for three days. Wild-type *Anabaena* was used in parallel to control endogenous fluorescence signals. The GFP fluorescence was visualized and quantified and the ratio to the background signal in wild-type *Anabaena* is presented.

GFP fluorescence above background was observed after seven or ten days of iron starvation in filaments of AFS-I-P*hutA1,* but not in AFS-I-P*hutA2* (**Figure 3B**; bars one and two). The addition of 10 µM protoporphyrin IX did not alter the GFP-fluorescence level in the two mutant strains (bar three). However, the signal in AFS-I-P*hutA1* was reduced when cells were grown in iron-free medium supplemented with 10 µM heme or in standard medium (bars four and five). These observations indicate that AFS-I-P*hutA1* is upregulated under iron starvation and that the induction of expression is iron and not backbone dependent, because protoporphyrin IX does not repress expression despite heme and protoporphyrin IX have an almost identical structure. The GFP fluorescence signal in AFS-I-P*hutA2* was almost not detectable under any condition. Only after the addition of heme at a higher concentration (30 µM) an increase of the GFP signal was observed (bar five).

The velocity of *hutA1* induction in the mutant AFS-I-P*hutA1* was also determined. The strains were grown in YBG11 medium and then exposed to iron starvation by transfer into iron-free YBG11 medium to monitor the GFP signal. At the time of inoculation in YBG11-Fe medium, no GFP signal was detectable (**Figure 3C** and **3D**, day 0). After two and four days of growth in the absence of iron, a comparable GFP signal was observed (second and third panels). This shows that starvation occurs faster than expected from typical chlorophyll *a* monitoring [35].

The genetic approach was complemented by the analysis of the mRNA abundance by qRT-PCR in the wild-type*. Anabaena* was pre-starved for 21 days and then transferred to either YBG11 medium, iron-depleted medium or iron-depleted medium supplemented with 30 µM heme. After seven days, mRNA was analyzed and the expression was normalized to *rnpB* levels and the level of the gene in cells grown in YBG11 medium. The high expression of *hutA1* observed during continuous iron starvation in comparison to growth in YBG11 medium (**Figure 3E**, blue) parallels the observation with the mutant strain (**Figure 3B**). Similarly, the transcript abundance of *hutA1* was reduced in the presence of heme when compared to iron-depleted medium, although the expression was still enhanced compared to the one found in cells grown in standard medium (**Figure 3E**, orange bar). For *hutA2*, qRT-PCR analysis confirmed the results observed using the GFP reporter as well. A similar transcript abundance of *hutA2* was found when cells were grown in iron-depleted or iron-containing medium (**Figure 3E**, blue bar). In the presence of heme, the mRNA level of *hutA2* was enhanced when compared to that of cells grown in YBG11 medium (**Figure 3E**, orange bar).

Taken together, our results show that the expression of *hutA1* is clearly dependent on the level of iron supply, which confirms previous observations [30, 35, 39, 40]. Its induction occurs shortly after sensing of iron starvation, which is more rapid than the one seen for the schizokinen sensing system [35, 36]. Moreover, its induction can in part be hampered by addition of heme, but not by addition of the protoporphyrin IX, which supports the dependence on iron rather than on the heme backbone (**Figure 3**). In contrast, the expression of *hutA2* appears to be independent of iron starvation, which parallels previous reports [30, 40]. However, *hutA2* expression is dependent on elevated levels of heme and, interestingly, is also regulated by FurA [38]. Thus, the increase of expression of *hutA2* might be related to the observed heme dependence of FurA in *Anabaena* [41] and, therefore, FurA might serve as a sensor of heme availability for *hutA2*.

### The function of HutA1 and HutA2 is related to heme uptake

**Figure 4:**
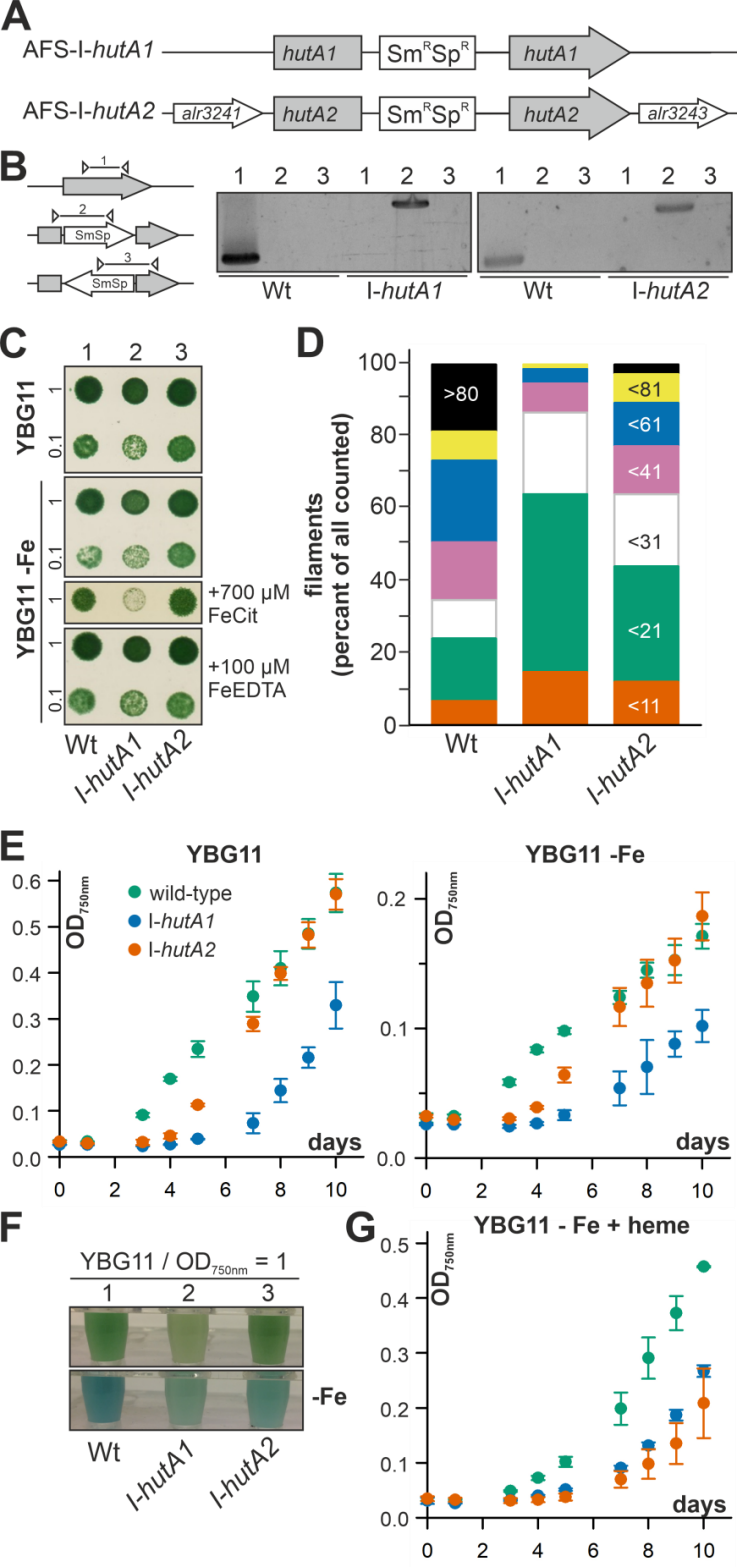
FIGURE 4: Role of *alr2153* and *alr3242* in heme uptake. **(A)** Strategy model for plasmid insertion in *Anabaena* genome to generate AFS-I-*hutA1* and AFS-I-*hutA2.* **(B)** Genomic DNA isolated from *Anabaena* wild-type, AFS-I-*hutA1* and AFS-I-*hutA2* was used for PCR with gene-specific primers (lane 1) or plasmid-specific and gene-specific right (lane 2) or left internal primers (lane 3). **(C)** 10 µl of a culture of *Anabaena* wild-type (lane 1), AFS-I-*hutA1* (lane 2) or AFS-I-*hutA2* (lane 3) with a density OD_750nm_ of 1 or 0.1 as indicated were spotted on solid YBG11 with (upper panel) or without iron (panel 2-4) supplemented with Fe-citrate (panel 3) or Fe-EDTA (panel 4). Representative images of plates after incubation for seven days are shown. **(D)** The three strains were grown in liquid YBG11 supplemented with 10 mM NaHCO_3_ and bubbled with CO_2_-enriched air (1% CO_2_ v/v). The filament length was counted for 75 filaments in three independent cultures. The bin size is indicated on the right bar. **(E)** Growth of the three strains in YBG11 (left) or YBG11 –Fe (right) was monitored at OD_750nm_ after inoculation with a starting OD_750nm_ of 0.02. The color code is given on the left plot. **(F)** The three strains were grown for seven days in YBG11 (top) or YBG11 –Fe (bottom) and cultures at OD_750 nm_ = 1 are shown. **(G)** Growth of the three strains in YBG11 –Fe with 30 µM heme was monitored at OD_750nm_ after inoculation with a starting OD_750nm_ of 0.02. The color code is given in (E).

*Anabaena* is able to take up and utilize exogenous heme (**Figure 1, 2**) and the expression of *hutA1* and *hutA2* appears to be iron and heme dependent (**Figure 3**). To gain insight into their function, plasmid insertion mutants previously generated and annotated as AFS-I-*hutA1* and AFS-I-*hutA2* (**Figure 4A**; [36]) were analyzed*.* Segregated colonies were isolated for both mutants (**Figure 4B**), indicating that the genes are not essential. It is worth mentioning that we were unable to create a double mutant, even when grown in normal media.

The growth of the single mutants on solid YBG11 medium was not reduced when compared to wild-type (**Figure 4C**, upper panel). The growth of the mutant strains was also not drastically altered by removal of iron or replacement of the iron source by Fe-EDTA (**Figure 4C**, second and fourth panel). Only when the iron source was replaced by iron citrate, AFS-I-*hutA1* showed a significant reduction of growth on solid medium (**Figure 4C**, third panel).

Mutants of outer membrane proteins or outer membrane biogenesis factors often show an altered membrane integrity or filament length. Hence, the filament lengths of *Anabaena* wild-type and mutants grown in bubbled YBG11 was determined. About 50% of all filaments of wild-type *Anabaena* contain more than 60 cells (**Figure 4D**, first bar). In contrast, two thirds of all filaments of AFS-I-*hutA1* are composed of 20 or less cells (**Figure 4D**, second bar). The filaments of AFS-I-*hutA2* are in general longer than that of AFS-I-*hutA1* but shorter than that shown by the wild-type, as more than 50% of all filaments contain less than 31 cells (**Figure 4D**, third bar). The filament length reduction could be related to a general defect of the outer membrane because mutants with enhanced outer membrane permeability show shorter filaments [42]. Alternatively, it could be related to a reduced iron uptake capacity. On the one hand, it was documented that filament length was dependent on iron availability, e.g. for *Lyngbya majuscule* [43]. On the other hand, it is suggested that the filament length is optimized toward light perception and, thus, photosynthetic capacity [44], which would be reduced by iron limitation (e.g. [3, 45].

The difference of the filament length prompted the analysis of the general growth behavior in liquid medium. In standard YBG11 medium AFS-I-*hutA2* shows an initial growth delay but subsequently a similar growth as found for the wild-type (**Figure 4E**, left plot, green and orange). In contrast, AFS-I-*hutA1* exhibits a general growth delay (**Figure 4E**, left plot, blue). All strains show a growth reduction in medium without iron when compared to normal growth conditions, but with the same behavior of the mutants as seen for YBG11 (**Figure 4E**, right plot). However, AFS-I-*hutA1* exhibits a pale phenotype under both conditions (**Figure 4F**). Moreover, in the absence of iron AFS-I-*hutA2* shows a pale phenotype in comparison to the wild-type as well (**Figure 4F**, bottom panel). When heme is the only iron source, the wild-type is able to recover growth close to standard medium levels (**Figure 4G**, green vs. **Figure 4E**, left plot, green), while both mutants show a reduced growth (**Figure 4G**). This clearly supports the bioinformatics classification of HutA1 and HutA2 as heme transporters.

**Figure 5:**
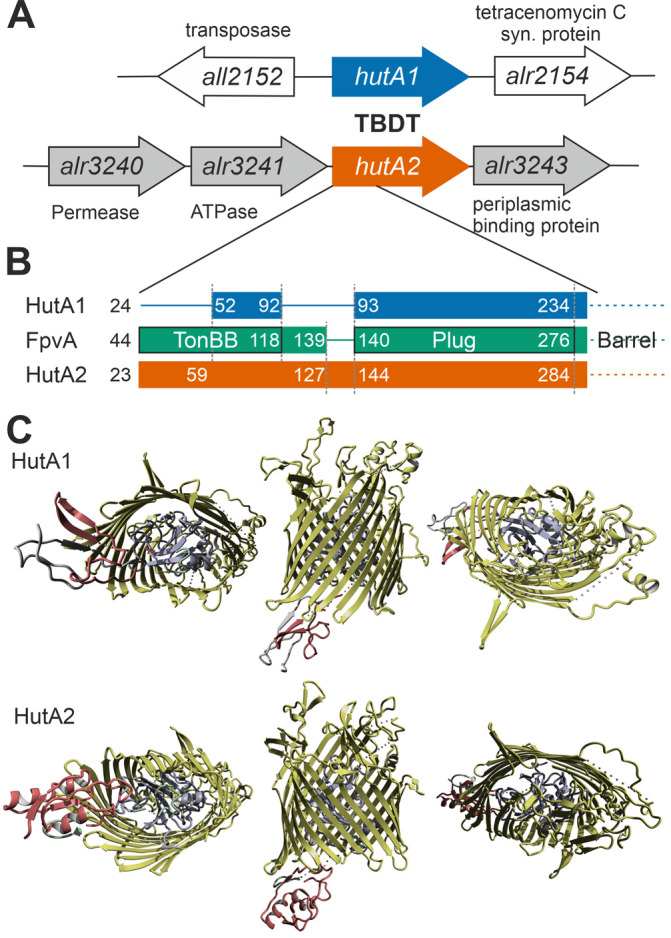
FIGURE 5: Comparison of HutA1 and HutA2 properties by bioinformatics. **(A)** Model of the genomic region surrounding *hutA1* and *hutA2* sequence. **(B)** Bars show the alignment of the N-terminal domain containing the TonB box and the plug domain between HutA1, HutA2 and FpvA (PDB: 2IAH). Numbers indicate the amino acid position of the full-length sequence. **(C)** Structural model of HutA1 and HutA2 created by structural modeling as described in Materials and Methods.

Remarkably, the gene coding for HutA2, but not the one coding for HutA1, is located in close genomic proximity to three genes putatively coding for a plasma membrane-localized ABC transporter (*alr3240, alr3241* and *alr3243*; **Figure 5A**). This putative transporter remains to be characterized, but comparison to other pathogenic Gram-negative bacteria or marine bacteria such as *Microscilla marina* suggest a function as a second part of the heme transport system for the three proteins [17, 46].

Moreover, based on structural modeling using the structure that was obtained as best hit (ferripyoverdine receptor FpvA; PDB: 2IAH; [47]), HutA1 and HutA2 contain a TonB box (TonBB) of different length (**Figure 5B**)*.* HutA1 has a very short TonBB and the linker between TonBB and the plug domain is absent. In turn, HutA2 has a prolonged linker domain between TonBB and the plug domain (**Figure 5B**)*.* Thus, structural modelling allows the prediction of a typical heme-transporting TBDT with a standard plug domain for HutA2 (**Figure 5C**). The TonBB is predicted to form a helical structure, with two β-sheets but at the N-terminus and, thus, distant from the plug domain. HutA1 is also modelled as a typical TBDT including the plug domain, but the TonBB is modelled as a β-sheet structure close to the plug domain (**Figure 5C**). Hence, it is tempting to speculate that the two TBDTs are energized by a different mechanism, which would be consistent with a different phenotype of the two mutants. Whether the different mode of energizing is related to the interaction with different TonB proteins [48] remains to be established.

## DISCUSSION

*Anabaena* is able to utilize heme for growth, because external heme can compensate for the absence of other iron sources (**Figure 1**) and the loss of function of either one of the heme transporter results in growth delay when heme is the only iron source (**Figure 4**). The internalized heme can either be used as metabolite or by its decomposition as iron source. The reduced expression of *hemH* in the presence of external heme (**Figure 2**) supports the first part of the hypothesis. The enhanced expression of *pebA* after addition of heme in comparison to addition of iron (**Figure 2**) supports the second part of the notion. The latter is further consistent with the observed repression of *hutA1* expression in the absence of iron by addition of heme, but not by addition of iron-free protoporphyrin IX (**Figure 3**). Moreover, the chlorophyll *a* level recovers after heme addition (**Figure 1**), which is consistent with both mechanisms. The velocity of iron removal from heme appears to be fast because *hutA1* expression in the absence of iron is reduced 24 hours after heme addition (**Figure 3**).

The iron starvation-dependent expression of *hutA1* (**Figure 3**) [30, 35, 39, 40] suggests that the encoded protein is required for the optimization of the uptake of xenosiderophores under iron limiting conditions. Consistent with this notion, in the presence of high concentrations of heme (30 µM) the expression of this gene is reduced likely to downregulate the rate of uptake (**Figure 3**). In contrast, *hutA2* appears to be expressed at a basal level with a putative induction by heme supplementation (**Figure 3**). This could suggest that the main function of HutA2 is the uptake of heme as nutrition. The enhanced expression in the presence of high concentrations of heme are consistent with this notion, as the protein is only produced when the nutrition is available. Consistent with an in part distinct function of the two proteins, both mutants display some phenotypic differences. For example, AFS-I-*hutA1*, but not AFS-I-*hutA2*, shows a pale phenotype under normal growth conditions, and the growth delay of AFS-I-*hutA1* is more pronounced than that seen for AFS-I-*hutA2* (**Figure 4**). Most remarkably, AFS-I-*hutA1* is strongly impaired in growth in the presence of iron citrate, while AFS-I-*hutA2* shows a growth comparable to wild-type (**Figure 4**). Consistent with a differential functionality, the two TBDTs HutA1 and HutA2 show distinct structural properties as discovered by bioinformatics analysis (**Figure 5**). However, the function of the two proteins is in part overlapping, as both mutants show shorter filaments when compared to the wild-type under normal growth conditions and a growth phenotype related to iron and heme availability (**Figure 4**). In addition, we were unable to generate a double mutant, which on the one hand is consistent with a functional overlap, and on the other hand suggests an additional function of the TBDTs even. Thus, we can conclude that the *Anabaena* genome encodes for two outer membrane proteins with capacity to transport heme, while both proteins might serve additional functions. In addition, the presence of HutA-like proteins in cyanobacteria is not limited to *Anabaena*, as previous bioinformatics analysis identified sequences with similar features encoded in the genome of some cyanobacteria of the order Chroococcales, but not of the order Gloeobacterales [30]. At the time of the analysis genomic sequences of cyanobacteria of the order Oscillatoriales and Prochlorales were not available. An initial analysis, however, documents that in some species of these orders as well as in other species then *Anabaena* in the order Nostocales HutA-like sequences can be detected (**Table 1**). Worth mentioning, in the other species analyzed previously and in here only a single gene coding for a HutA-like protein was detected, and in all analyzed orders species were identified in which a HutA-like sequence could not be identified (**Table 1**, [30]). We conclude that heme transport might not be restricted to *Anabaena*, but at the same time is not specific to a cyanobacterial order and thus might be related to the habitat of origin, a notion which needs to be challenged in the future.

**Table 1.** Identified sequences with similarity to HutA1 or HutA2 encoded by the genome of the cyanobacteria.

**Order**	**Species**	**HutA proteins**		**Other TBDTs**		
		**HutAl**	**HutA2**	**SchT**	**lutAl**	**ALR3310**
Nostocales	Fischerella thermalis M66 A2018 004	MBF2059227 (0.0)	MBF2059227 (3.00e-146)	X	MBF2061156 (1.41e-72)	X
Prochlorales	Candidatus Paraprochloron terpiosi SP5CPC1	MBC6419424 (3.93e-180)	MBC6419424 (0.0)	MBC6419424 (1.13e-17)	MBC6419424 (9.97e-20)	MBC6419424 (1.25e-07)
Oscillatoriales	Lyngbya confervoides BDU141951	MCM1982914 (8.19e-175)	MCM1982914 (7.66e-155)	X	X	X
Oscillatoriales	Oscillatoria sp. CS-180	MDB9525528 (3.69-15s)	MDB9525528 (8.55e-151)	X	X	MDB9529641 (1.08e-07)
Oscillatoriales	Trichodesmium sp. MAG_R04	MCL2924212 (0.0)	MCL2924212 (7.12e-127)	X	X	X

Shown is the order, the name of the species, the accession number of the orthologue confirmed by reversed blast analysis and the e-value of the similarity.

## MATERIAL AND METHODS

### Bacterial strains and growth conditions

*Anabaena* sp. (also known as *Nostoc* sp.) PCC 7120 wild-type and mutant strains were grown photoautotrophically under constant light conditions (70 µmol photons m^−2^s^−1^) at 30°C. Cultures were grown in liquid YBG11 [49] medium (shaking: 100 rpm) or on solidified YBG11 medium prepared by addition of 1% Bacto agar (Otto Nordwald GmbH, Hamburg, Germany). When indicated, cultures were supplemented with 10 mM NaHCO_3_ and bubbled with CO_2_-enriched air (1% CO_2_ v/v). Mutant strains were supplemented with streptomycin and spectinomycin (final concentration: 5 µg ml^−1^ each). *Anabaena* strains used in this study are listed in **Table 2**, and previously created strains are described in [36].

**Table 2.** Strains used in this study.

Strain	Resistance	Genotype	Properties	Source
*Anabaena sp.* PCC 7120			Wild-type	C. P. Wolk
AFS-I-*hutA1*	Sm, Sp	*alr*2153::pCSV3	Gene interruption by pCSV3 plasmid	[36]
AFS-I-*hutA2*	Sm, Sp	*alr*3242::pCSV3	Gene interruption by pCSV3 plasmid	[36]
AFS-I-*PhutA1*	Sm, Sp	*Palr*2153-*gfp*	N-terminal GFP fusion	This work
AFS-I-*PhutA2*	Sm, Sp	*Palr*3242-*gfp*	N-terminal GFP fusion	This work

AFS: *Anabaena* Frankfurt Schleiff; Sm: streptomycin; Sp: spectinomycin.

For iron starvation, all flasks were treated with hydrochloric acid (10%) and extensively rinsed with ddH_2_O. Pre-starvation was obtained by growth for 21 or seven days in the absence of an iron source. Chlorophyll *a* content was determined to assure the starvation status [35]. For growth rate determination, cultures were harvested and washed with iron-depleted YBG11. Cells were inoculated at OD_750 nm_ = 0.02. OD_750 nm_ was recorded once per day. For phenotyping on solid medium, cultures were grown for 5-7 days in liquid YBG11 and 5 µl of cell suspension (OD_750_ = 1) were spotted on YBG11 plates of indicated composition. Growth was monitored for 7 days.

Fragmentation of filaments was analyzed by counting the number of cells per filament after 3 days of growth (inoculation OD_750 nm_ = 0.02) in bubbled YBG11 medium. Microscopy was performed after 3 days of growth and cells of 75 filaments per strain were counted as previously described [42].

### Expression analysis

RNA was isolated from strains that had been grown for 7 days in standard YBG11 medium, iron-depleted YBG11 medium and iron-depleted YBG11 medium supplemented with 30 µM heme. RNA was extracted with TRIzol (Thermo Fisher Scientific, Waltham, Massachusetts, USA) and treated with DNaseI as described [40]. cDNA synthesis was done with RevertAid Reverse transcriptase from Thermo Fisher Scientific.

qPCR was performed with a StepOnePlus Cycler (Thermo Fisher Scientific) and with PowerUp SYBR Green Master Mix (Applied Biosystems, Waltham, Massachusetts, USA). The oligonucleotide pairs chosen for this study were previously tested to yield a single product and the obtained values were normalized to the expression values obtained for *rnpB* [40, 50]. The ΔΔct was calculated by normalization to the Δct of the wild-type [50].

### Genetic procedures

Genomic DNA from *Anabaena* was isolated from 50-ml shaking cultures [51]. To generate the strains AFS-I-P_*alr2153*_ and AFS-I-P_*alr3242*_ (**Table 2**) 487- and 316-bp fragments of the upstream region of *alr2153* and *alr3242*, respectively, including the start codon of the respective gene, were amplified by PCR on genomic DNA with specific oligonucleotides (**Table 3**) containing ClaI/EcoRV restriction sites. The amplified fragments were cloned into the vector pGEM-T Easy (Promega GmbH, Walldorf, Germany; **Table 4**) for sequencing.

**Table 3.** Oligonucleotides used in this study.

Purpose	Name	Sequence
Cloning	hutA1-PGFP-fw	GGATAAATGTCATCGATGGAGG
	hutA1-PGFP-rv	AAGCCGATATCCATCTGATC
	hutA2-PGFP-fw	GATTCAATATCGATTTTGTCGG
	hutA2-PGFP-rv	GAACAAATAGATATCCATGAATTCTCC
Segregation analysis	hutA1-S-fw	GCACCACAGACACCAATG
	hutA1-S-rv	GGTGAAATGGCAGAATCG
	hutA2-S-fw	GTCGGAACATTTGTCCAAG
	hutA2-S-rv	GGAGAAAGTGCAGATGTGG
	hutA1-PGFP-S-fw	GCCAGTCAAGATTGCTAGCC
	hutA1-PGFP-S-rv	GCCAATCAGCACTTCTATGGG
	hutA2-PGFP-S-fw	CCAGGGATTACGATTTTAACC
	hutA2-PGFP-S-rv	CGTTTGGTAATGTTCCATCTCC
	pCSV3-R	CTGATGCCGCATAGTTAAGCC
	GFP-4	CAAGAATTGGGACAACTCC
RT-PCR	rnpB-RT-fw	AGGGAGAGAGTAGGCGTTGG
	rnpB-RT-rv	GGGTTTACCGAGCCAGTACC
	all4365-RT-fw	GGTACGAGCATCTAGGCGG
	all4365-RT-rv	GGAAATGTACCTCAAGCGC
	all4480-RT-fw	GAAGGTGATTTGGGGAATG
	all4480-RT-rv	GCTTTACCTTGGTGGGCTG
	all3751-RT-fw	CCACTACGGAGGATTACGG
	all3751-RT-rv	GCCATCGCCTGTAAATAACC
	all0287-RT-fw	TGCGAAAATTCAGCTACAATCC
	all0287-RT-rv	TCTGCAAGGGTGGTTTACCG
	alr0115-RT-fw	CTTTGGCAGAGGCGATTAGG
	alr0115-RT-rv	GATCGCCAAAGCTTCCACAC
	alr3707-RT-fw	GTTTGGCATCAGCACCTG
	alr3707-RT-rv	GGGTAGGGTGCGGTCTAAG
qPCR	rnpB-qRT-fw	GTAGGCGTTGGCGGTTG
	rnpB-qRT-rv	CACTGGACGTTATCCAGC
	hutA1-qRT-fw	CAGTGAATCGCGGTGAAGTG
	hutA1-qRT-rv	GTCGTCTCCGACAGTGTAAC
	hutA2-qRT-fw	GCTATGGTGCTACTCGTTTG
	hutA2-qRT-rv	CGCGTCACTTCCTAATACTG

**Table 4.** Plasmids used in this study.

Plasmid	Resistance	Insert	Purpose	Reference
pGEM-T	Amp		Cloning	
pCSV3	Sp^R^/Sm^R^		Cloning	[61]
pCSEL21	Amp^R^	*gfp-mut2*		[52]
pCSV3-hutAl	Sp^R^/Sm^R^	Internal fragment of *alr*2153	Generation of singlerecombinant mutants	[36]
pCSV3-hutA2	Sp^R^/Sm^R^	Internal fragment of *alr*3242		[36]
pCSEL21-hutAl	Sp^R^/Sm^R^	Internal fragment of *alr*2153	Generation of promoter-*gfp* fusions	This work
pCSEL21-hutA2	Sp^R^/Sm^R^	Internal fragment of *alr*3242		This work

Sp: spectinomycin; Sm: streptomycin; Amp: ampicillin.

After confirming the right sequence, the fragments were restricted and cloned into pCSEL21, which contains the *gfp* ORF [52]. Digestion with EcoRI resulted in the excision of the fusion fragment, which was then ligated to pCVS3 and conjugated into wild-type *Anabaena*. Conjugal transfer of plasmids into *Anabaena* was done as described [53, 54]. Incorporation of the fusion fragment into the *Anabaena* genome was confirmed by PCR.

*Escherichia coli* DH5α, HB101 and ED8654 were used for plasmid constructions as well as conjugations into *Anabaena* [55, 56].

### Confocal microscopy

Microscopy was in general performed as described in [57]. In brief: 50 µl of *Anabaena* wild-type or mutant strain samples from liquid cultures were placed on top of BG11 solid medium and visualized using a 63X 1.40 oil immersion objective attached to the confocal laser-scanning microscope Zeiss LSM 780. GFP was excited with an argon ion laser at 488 nm irradiation. Fluorescent emission for GFP imaging and cyanobacterial autofluorescence was collected between 500-540 nm and 630-700 nm, respectively.

### Quantification of GFP fluorescence

The GFP fluorescence signal was quantified in two different ways. (i) Experiments shown in Figure 3B were performed in a Tecan Spark 10 M plate reader with the excitation wavelength at 488 nm and the GFP fluorescence emission measurement at 533 nm. The optical density of the respective cultures was determined at 750 nm. A fluorescein solution (final concentration 1 ng/ml) was used as reference. 200 µl of each culture were measured in a black 96-well microplate with a clear bottom (Greiner Bio-One GmbH, Frickenhausen, Germany). The GFP fluorescence of each sample was normalized to the fluorescence of the fluorescein reference and to the optical density of each sample. The signal of the mutant was normalized to the wild-type. (ii) The quantification of the GFP fluorescence determined by confocal microscopy presented in Figure 3D was performed with the ZEN software by Zeiss (Carl Zeiss Microscopy GmbH, Oberkochen, Germany). A cross section of selected cells in the images was taken. The values in the GFP channel were averaged to get a final GFP count. The autofluorescence channel was used as control. Final GFP count of the mutant was normalized to wild-type and displayed.

### Structure prediction

Structure prediction was based on Phyre2 [58]. The sequences of HutA1 (Alr2153) and HutA2 (Alr3242) were used as query with a secondary structure prediction to create a hidden markov model based on a multiple sequence alignment. Based on a search against known structures the backbone was modelled using the structure of the best hit (ferripyoverdine receptor FpvA; PDB: 2IAH; [47]) followed by loop and sidechain modelling with Phyre2. The alignment coverage was 93% (HutA1) and 97% (HutA2) with a confidence of 100% and a sequence identity of 16% (HutA1) and 18% (HutA2). YASARA including the structural alignment module MUSTANG [59] was used to define the structural domains (TonB box, intramolecular domain and barrel domain) and to create representations.

### Sequence analysis

The protein sequences for Alr3242 (HutA2), Alr2153 (HutA1), Alr0397 (SchT), Alr2581 (IutA2) and All3310 [30, 34–36] were used for a best-blast-hit search on the level of the different taxonomic orders (Nostocales, Prochlorales and Oscillatoriales). From the 100 top-hits within each lineage sequences were selected from up to five different species for reciprocal best-BLAST-hit search against the translated genome of *Anabaena* [60]. Examples of species in which HutA-like sequences were identified are listed in **Table 1**.

## SUPPLEMENTAL MATERIAL

Click here for supplemental data file.

All supplemental data for this article are available online at www.microbialcell.com/researcharticles/2024a-graf-microbial-cell/.

## Abbreviations:

GFP: – green fluorescent protein,
TBDT: – TonB-dependent transporter.
